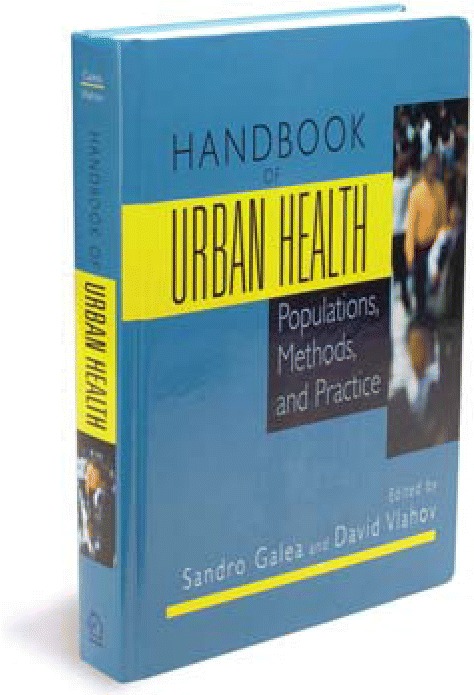# Handbook of Urban Health: Populations, Methods, and Practice

**Published:** 2006-01

**Authors:** Tord Kjellstrom

**Affiliations:** Tord Kjellstrom trained in medicine and engineering at universities in Stockholm, Sweden. He has more than 30 years experience in environmental and occupational health, primarily as an academic teacher and researcher in Sweden, New Zealand, and Australia. In addition, he was an environmental epidemiologist for 12 years at the World Health Organization. He is currently Visiting Professor at the Swedish National Institute of Public Health and Visiting Fellow at the National Centre for Epidemiology and Population Health, Australian National University.

Edited by Sandro Galea and David Vlahov

New York:Springer, 2005. 599 pp. ISBN- 0-387-23994-4, $89.95

The world is undergoing major urbanization. Within 25 years, more than half the world’s population will be living in urban areas, and in this period approximately 1 million people will be added to city populations each week. Urban health is thus significant for population health, and this handbook provides a timely review of the issues involved.

This book takes a broad view of urban health, emphasizing urban social factors important to population health. The editors successfully bridge urban health inquiry and public health practice by combining descriptions of issues in urban health, methods used in urban health studies, and examples from practitioners. The authors of the 29 chapters come from different professional backgrounds, primarily in North America, but those from developing countries add a global flavor.

Part 1, “Populations,” includes 11 chapters on health aspects of different socioeconomic groups in cities and different age groups, describing time trends and geographic differences. Special issues for minority groups are reviewed, and each chapter provides a wealth of up-to-date research references.

In Part 2, “Methods,” 10 chapters present methods in anthropology, epidemiology, demography, sociology, environmental health, and economic analysis. Each chapter provides relatively detailed descriptions, some including detailed mathematical formulas for analyzing data. Although this handbook can present only part of the knowledge required to be fully conversant with any of the methods, these chapters give a good overview of methods available in urban health. One limitation lies in the examples used to describe the methods. For example, the environmental health chapter deals almost exclusively with urban air pollution while other important urban health hazards, such as community noise, are barely mentioned.

Part 3, “Practice,” includes excellent examples of the broad approach to urban health used in the Healthy Cities movement as well as more focused examples from a local health department level. Legal issues and suggestions for teaching urban health are also presented. The editors note that success in interventions that target proximal determinants of health depends on more upstream laws and regulations. Promoting health in cities requires an appreciation of the multiple levels of determinants that shape population health, and this handbook is a good starting point for such appreciation.

In handbooks that cover a multitude of fields and examples, some issues are not given the space that they may deserve. Here, apart from the limited range of environmental health issues presented, the book rarely mentions injuries and their more proximal determinants. Living and working in cities almost invariably require daily transport, and the risks involved are surely urban health issues. Neither is much attention given to workplace factors and health, even though cities serve as magnets for both people and different types of modern workplaces. A variety of occupational health hazards, including psychosocial factors as well as traditional chemical and physical hazards, are prominent determinants of population health in urban areas.

Nevertheless, the editors intended the handbook to form one step toward the systematic study of urban health. They have succeeded by giving readers a thorough view of the social factors involved. The handbook is an excellent resource for students, researchers, teachers, and practitioners in urban health. The aspects of urban health given less prominence in this volume may be a suitable focus of a companion volume at a later stage. Further study of urban health situations and determinants is required to meet the challenges of global urbanization during this century. This handbook is a valuable contribution.

## Figures and Tables

**Figure:**